# Adipose-inflammatory factor profiles in children with metabolically healthy obesity and their correlation with NAFLD severity

**DOI:** 10.3389/fped.2026.1767970

**Published:** 2026-04-10

**Authors:** Jing Li, Hongyun Shi, Qiaoheng Xie, Ping Zhang, Xiangyun Liu

**Affiliations:** 1 The Second Affiliated Hospital, Department of Pediatrics, Hengyang Medical School, University of South China, Hengyang, China; 2 The Second Affiliated Hospital, Department of Medical Affairs, Hengyang Medical School, University of South China, Hengyang, China

**Keywords:** adipose-inflammatory factors, correlation, metabolically healthy obesity, metabolically unhealthy obesity, NAFLD

## Abstract

**Objective:**

To compare adipose-inflammatory factor profiles between children with metabolically healthy obesity (MHO) and metabolically unhealthy obesity (MUO), and analyze their associations with non-alcoholic fatty liver disease (NAFLD) severity in MHO.

**Methods:**

This retrospective study included 500 obese children (162 MHO, 338 MUO) and 162 metabolically healthy lean (MHL) controls. Anthropometric, metabolic parameters, and serum levels of key adipose-inflammatory factors (including adiponectin, leptin, resistin, RBP-4, PGRN, TNF-α, IL-6, and CCL2) were compared. ROC curve analysis was used to evaluate diagnostic efficacy of adipose-inflammatory factors for differentiating phenotypes. NAFLD prevalence was assessed, and relationships of adipose-inflammatory factors with NAFLD activity score (NAS) and steatosis, activity, and fibrosis (SAF) score in MHO children with NAFLD were analyzed by Spearman's correlation analysis.

**Results:**

Metabolic parameters and adipose-inflammatory factor levels (leptin, resistin, RBP-4, PGRN, TNF-α, IL-6, CCL2) were significantly higher in MHO than MHL, and further elevated in MUO, while adiponectin showed an inverse trend (all *P* < 0.05). These factors demonstrated good to excellent diagnostic value for distinguishing MHL from both obese phenotypes (AUC range: 0.695–0.894), and moderate value for distinguishing MHO from MUO (AUC range: 0.636–0.740; all *P* < 0.001). NAFLD prevalence was 29.01% in MHO vs. 46.15% in MUO (*P* < 0.001). In MHO children with NAFLD, adiponectin levels correlated negatively with NAS and SAF score (*r* = −0.668, −0.641), whereas all other factors showed positive correlations (*r* = 0.468–0.681, all *P* < 0.001).

**Conclusion:**

MHO children exhibit dysregulation of adipose-inflammatory factors and a considerable risk for NAFLD. These factors, especially adiponectin and leptin, effectively discriminate metabolic phenotypes and correlate with liver injury severity in MHO, suggesting their potential utility as early biomarkers and therapeutic targets.

## 1 Introduction

Childhood obesity has become one of the most critical public health challenges globally, with its prevalence escalating dramatically in recent decades (1). Obesity is not only an aesthetic concern but also a significant risk factor for various metabolic disorders, often accompanied by insulin resistance (IR), dyslipidemia, hypertension, and non-alcoholic fatty liver disease (NAFLD) (2, 3). However, previous research has revealed substantial metabolic heterogeneity among obese individuals (4). This metabolic heterogeneity has led to a refinement of the obesity concept, classifying it into two phenotypes based on metabolic health: metabolically healthy obesity (MHO) and metabolically unhealthy obesity (MUO). MHO refers to individuals who meet the criteria for obesity but maintain normal metabolic parameters such as blood pressure, blood glucose, and lipids, with relatively preserved insulin sensitivity. In contrast, MUO denotes obesity coexisting with one or more components of metabolic dysregulation (5). The estimated prevalence of MHO among obese adults ranges from 6% to 40%, but reported rates in children and adolescents vary considerably due to the lack of uniform diagnostic criteria (6). Historically, MHO was perceived as benign, and its long-term health risks were not fully recognized. However, growing evidence suggests that MHO may represent a relatively stable or transient metabolic adaptation phase. While the risks of cardiovascular events and type 2 diabetes are lower in individuals with MHO than in those with MUO, these risks remain significantly higher than in their metabolically healthy lean (MHL) counterparts (7). Notably, the risk of NAFLD, one of the most common hepatic complications of obesity, in children with MHO remains unclear. The NAFLD spectrum ranges from simple hepatic steatosis to non-alcoholic steatohepatitis, potentially progressing to fibrosis, cirrhosis, and even hepatocellular carcinoma (8). Disease progression may be more rapid in pediatric NAFLD and is strongly linked to end-stage liver disease and cardiovascular mortality in adulthood (9). Therefore, a deeper investigation into the risk and underlying mechanisms of NAFLD in MHO children is crucial for early identification of high-risk individuals and the implementation of precise interventions.

Obesity-related metabolic disorders are closely associated with a state of chronic low-grade inflammation. Adipose tissue is not merely an energy storage depot but an active endocrine organ secreting various adipokines and inflammatory cytokines, collectively termed adipose-inflammatory factors, which play a central role in regulating energy metabolism, insulin sensitivity, and inflammatory responses. Among these, adiponectin, a protective factor with insulin-sensitizing and anti-inflammatory properties, is typically reduced in obesity (10). Conversely, leptin, resistin, retinol-binding protein 4 (RBP-4), progranulin (PGRN), and classical inflammatory cytokines such as tumor necrosis factor-alpha (TNF-α), interleukin-6 (IL-6), and monocyte chemoattractant protein-1 (CCL2) predominantly exert pro-inflammatory and pro-IR effects, with their levels often elevated in obesity (11–13). However, existing research predominantly focuses on adults, confirming significant associations of these factors with metabolic health and NAFLD progression (14, 15). Currently, systematic studies targeting children with MHO remain scarce. Several critical aspects are yet to be investigated: the expression profiles of key adipose-inflammatory factors, their potential differences compared to MHL children, and whether their dysregulation is linked to the development and progression of NAFLD.

Based on this background, this study aims to comprehensively evaluate the profile of adipose-inflammatory factors in MHO children compared to MHL and MUO children and to analyze the discriminatory efficacy of these factors for different metabolic phenotypes. Furthermore, focusing on the subgroup of MHO children with NAFLD, this study will investigate the correlations between these adipose-inflammatory factors and the severity of histological liver damage, assessed by the NAFLD activity score (NAS) and steatosis, activity, and fibrosis (SAF) score, to elucidate their potential role in liver disease progression in MHO children. The findings may provide important serological evidence for identifying “high-risk MHO” children in the early disease stages and offer theoretical support and potential targets for developing targeted early intervention strategies.

## 2 Materials and methods

### 2.1 Demographic characteristics

A retrospective analysis included 500 children with obesity diagnosed at our hospital's pediatric health clinic between January 2023 and July 2025. They were categorized into MHO (*n* = 162) and MUO (*n* = 338) groups based on metabolic status. A control group of MHL children (*n* = 162) was selected during the same period. MHL group: age: [10.00 (8.00, 12.00)] years; gender: 88 males (54.32%) and 74 females (45.68%); ethnicity: 156 Han (96.30%) and 6 ethnic minorities (3.70%); guardian education: primary school 4 (2.47%), junior high 22 (13.58%), senior high 83 (51.23%), and university 53 (32.72%). MHO group: age: [10.00 (8.00, 13.00)] years; gender: 84 males (51.85%) and 78 females (48.15%); ethnicity: 153 Han (94.44%) and 9 ethnic minorities (5.56%); guardian education: primary school 7 (4.32%), junior high 27 (16.67%), senior high 78 (47.15%), and university 50 (30.86%). MUO group: age: [11.00 (8.00, 13.00)] years; gender: 198 males (58.58%) and 140 females (41.42%); ethnicity: 322 Han (95.27%) and 16 ethnic minorities (4.73%); guardian education: primary school 9 (2.66%), junior high 48 (14.20%), senior high 154 (45.56%), and university 127 (37.57%). No statistically significant differences were observed in demographic characteristics among the three groups (*P* > 0.05), indicating comparability.

### 2.2 Diagnostic criteria

(1) MHO (5, 16–18): ① Body mass index (BMI) ≥ 95th percentile (for age and sex, applicable for age > 2 years); ② Systolic blood pressure (SBP) and diastolic blood pressure (DBP) < 90th percentile for age and sex; triglycerides (TG) < 1.70 mmol/L; high-density lipoprotein cholesterol (HDL-C) > 1.03 mmol/L; fasting blood glucose (FBG) < 5.6 mmol/L.

(2) MUO (5): Defined as obesity coexisting with at least one component of metabolic abnormality, including elevated blood pressure, dyslipidemia, hyperglycemia, or IR.

(3) NAFLD (19): Chronic hepatic steatosis occurring in children (≤18 years); other causes of hepatic steatosis must be excluded (e.g., alcohol consumption, genetic metabolic diseases, hepatotoxic drug use, infection, or malnutrition). To ensure consistency with the diagnostic criteria and terminology in use during the study enrollment period, the term “NAFLD” was adopted throughout this manuscript. Although the terminology of metabolic dysfunction-associated steatotic liver disease (MASLD) has recently been introduced and is gaining acceptance, this study retained the historical term solely for methodological and temporal consistency.

### 2.3 Inclusion criteria

① Age of 3–18 years; ② Meeting the diagnostic criteria for MHO, MUO, or NAFLD as defined in section 2.2; ③ First-time diagnosis of obesity at our hospital's pediatric health clinic; ④ Physical condition capable of tolerating study-related examinations and specimen collection procedures; ⑤ Complete clinical data required for the study; ⑥ Legal guardian signed the informed consent, and the study protocol was approved by the Ethics Committee of the Second Affiliated Hospital, Hengyang Medical School, University of South China (No. 2025095).

### 2.4 Exclusion criteria

① Secondary obesity due to endocrine disorders, genetic syndromes, or medications; ② Presence of liver diseases other than NAFLD that could cause hepatic steatosis or affect liver function, such as active viral hepatitis, autoimmune liver disease, drug-induced liver injury, genetic metabolic liver disease, or cholestatic disorders; ③ Severe systemic diseases, such as significant cardiac or renal disease, malignancy, active autoimmune disease, or acute/chronic infectious period; ④ Recent use (within a specified timeframe prior to the study) of medications potentially significantly affecting core study indicators (body weight, glucose/lipid metabolism, inflammatory status, hepatic fat), such as glucocorticoids, antipsychotics, immunosuppressants, lipid-lowering agents, or hypoglycemic drugs; ⑤ Diagnosed psychiatric illness/cognitive impairment; ⑥ Missing key clinical data or study-required laboratory measurements.

### 2.5 Observation indicators

(1) Anthropometric indicators: BMI was calculated as weight (kg)/height² (m²). Waist circumference (WC) was measured at the midpoint between the iliac crest and the lower rib margin using a non-elastic tape (precision 0.1 cm).

(2) Metabolic indicators: SBP and DBP were measured three times consecutively using an Omron electronic sphygmomanometer (HEM-7121, Japan), with the average recorded. For the analysis of FBG, TG, and HDL-C, 5 mL of fasting venous blood was collected into a coagulation-promoting tube. After standing at 37°C for 30 min and centrifugation at 3,000 rpm for 10 min (Centrifuge 5424R, Eppendorf, Germany), the serum was analyzed on an automatic biochemical analyzer (Cobas c702, Roche, Switzerland) via enzymatic colorimetric assays. Homeostatic model assessment-IR (HOMA-IR) was calculated as [fasting insulin (μU/mL) × FBG (mmol/L)]/22.5. Fasting insulin was measured by chemiluminescence (ADVIA Centaur XP, Siemens, Germany).

(3) Adipose-inflammatory factors: Adipokines included adiponectin, leptin, resistin, RBP-4, and PGRN. Inflammatory cytokines included TNF-α, IL-6, and CCL2. Fasting venous blood (10 mL) was collected into a coagulation-promoting tube, gently mixed, and centrifuged at 3,000 rpm for 15 min at 4°C. Serum was separated and stored for analysis. Corresponding enzyme-linked immunosorbent assay kits (R&D Systems, Abcam, BioLegend) were used following the manufacturers' protocols. Absorbance was measured using a microplate reader (Epoch2, BioTek, USA), and concentrations were calculated based on standard curves.

(4) NAFLD prevalence: The prevalence of NAFLD among children with different obesity phenotypes (MHO/MUO) was calculated.

(5) NAS (20): Based on liver biopsy histopathology (gold standard), NAS was assessed independently by two senior pathologists in a blinded manner, with arbitration by a third pathologist in case of disagreement. It consists of three components: ① steatosis (S0-3); ② lobular inflammation (LI0-3); ③ hepatocyte ballooning (B0-2). Total score ranges from 0 to 8, with higher scores indicating greater severity.

(6) SAF score (21): Based on liver biopsy histopathology (gold standard), SAF score was assessed independently by two senior pathologists in a blinded manner, with arbitration by a third pathologist in case of disagreement. It consists of three components: ① steatosis (S0-3); ② activity (A0-4), defined as the sum of scores for lobular inflammation (0-2) and hepatocyte ballooning (0–2); ③ fibrosis (F0-4). Total score ranges from 0 to 11, with higher scores indicating greater severity.

### 2.6 Statistical analysis

SPSS 27.0 was used for statistical analysis, and Prism 8.0.2 for graphing. Categorical data were presented as [*n* (%)] and analyzed using the *χ²* test or continuity-corrected *χ²* test. Normally distributed quantitative data were expressed as mean ± standard deviation ($\bar{x}$ ± *s*), with group comparisons performed using independent samples *t*-tests. Non-normally distributed quantitative data were expressed as *M* (*P25*, *P75*), with group comparisons performed using the *Mann–Whitney U-*test. Receiver operating characteristic (ROC) curves were used to evaluate the diagnostic efficacy of adipose-inflammatory factors in distinguishing MHL from MHO, MHL from MUO, and MHO from MUO. The Youden's index was calculated as sensitivity + specificity − 1. Spearman's correlation analysis was used to examine the relationship between adipose-inflammatory factor levels and NAS/SAF scores in MHO children with NAFLD. The significance level was set at *P* < 0.05.

## 3 Results

### 3.1 Comparison of anthropometric and metabolic indicators

BMI, WC, SBP, DBP, FBG, TG, and HOMA-IR were significantly higher in both the MHO and MUO groups compared to the MHL group, with all parameters higher in the MUO group than in the MHO group. HDL-C was lower in both MHO and MUO groups compared to the MHL group and was lower in the MUO group than in the MHO group (*P* < 0.05) (Table 1).

**Table 1:** Comparison of anthropometric and metabolic indicators among the three groups.

Indicator	MHL group (*n* = 162)	MHO group (*n* = 162)	MUO group (*n* = 338)
BMI (kg/m^2^)	17.65 (16.39, 19.12)	22.52 (20.80, 24.78)^a^	25.52 (23.35, 27.15)^a^^,^^b^
WC (cm)	63.00 (59.00, 68.00)	84.50 (79.00, 91.00)^a^	92.00 (85.00, 99.00)^a^^,^^b^
SBP (mmHg)	99.00 (97.00, 102.00)	109.00 (105.00, 112.00)^a^	118.00 (115.00, 121.00)^a^^,^^b^
DBP (mmHg)	64.00 (63.00, 65.00)	72.00 (68.00, 74.00)^a^	78.00 (76.00, 80.00)^a^^,^^b^
FBG (mmol/L)	4.82 (4.62, 5.02)	5.18 (5.02, 5.34)^a^	5.82 (5.48, 6.13)^a^^,^^b^
TG (mmol/L)	0.88 (0.76, 1.00)	1.22 (1.05, 1.41)^a^	2.14 (1.78, 2.43)^a^^,^^b^
HDL-C (mmol/L)	1.50 (1.36, 1.64)	1.21 (1.13, 1.29)^a^	0.95 (0.83, 1.08)^a^^,^^b^
HOMA-IR	1.89 (1.51, 2.23)	2.94 (2.39, 3.51)^a^	5.13 (4.14, 6.03)^a^^,^^b^

BMI, Body mass index; WC, Waist circumference; SBP, Systolic blood pressure; DBP, Diastolic blood pressure; FBG, Fasting blood glucose; TG, Triglycerides; HDL-C, High-density lipoprotein cholesterol; HOMA-IR, Homeostatic model assessment-insulin resistance.

^a^ *P* < 0.05 compared to the MHL group.

^b^ *P* < 0.05 compared to the MHO group.

### 3.2 Comparison of adipose-inflammatory factors

Leptin, resistin, RBP-4, PGRN, TNF-α, IL-6, and CCL2 levels were significantly higher in both MHO and MUO groups compared to the MHL group, with all levels higher in the MUO group than in the MHO group. Adiponectin levels were significantly lower in both MHO and MUO groups compared to the MHL group and were lower in the MUO group than in the MHO group (*P* < 0.05) (Table 2).

**Table 2:** Comparison of adipose-inflammatory factors among the three groups.

Factor	MHL group (*n* = 162)	MHO group (*n* = 162)	MUO group (*n* = 338)
Adiponectin (μg/mL)	11.93 (9.77, 13.73)	9.18 (7.95, 10.61)^a^	8.34 (7.17, 9.39)^a^^,^^b^
Leptin (ng/mL)	5.80 (4.61, 7.20)	8.82 (6.40, 11.22)^a^	12.60 (9.06, 15.69)^a^^,^^b^
Resistin (ng/mL)	8.36 (6.84, 10.01)	11.06 (8.89, 13.32)^a^	13.11 (10.01, 15.97)^a^^,^^b^
RBP-4 (μg/mL)	32.96 (27.48, 37.78)	39.61 (33.98, 45.79)^a^	46.25 (38.78, 53.36)^a^^,^^b^
PGRN (ng/mL)	45.96 ± 10.40	58.62 ± 13.49^a^	74.87 ± 20.03^a^^,^^b^
TNF-α (pg/mL)	6.93 (5.67, 8.20)	8.34 (6.77, 10.13)^a^	9.57 (7.67, 11.76)^a^^,^^b^
IL-6 (pg/mL)	2.25 (1.76, 2.71)	2.92 (2.24, 3.65)^a^	3.74 (2.48, 4.93)^a^^,^^b^
CCL2 (pg/mL)	187.13 ± 42.91	229.50 ± 57.80^a^	279.29 ± 83.94^a^^,^^b^

RBP-4, Retinol-binding protein 4; PGRN, Progranulin; TNF-α, Tumor necrosis factor-alpha; IL-6, Interleukin-6; CCL2, Monocyte chemoattractant protein-1.

^a^ *P* < 0.05 compared to the MHL group.

^b^ *P* < 0.05 compared to the MHO group.

### 3.3 Diagnostic efficacy of adipose-inflammatory factors in distinguishing MHL from MHO

**Figure 1:**
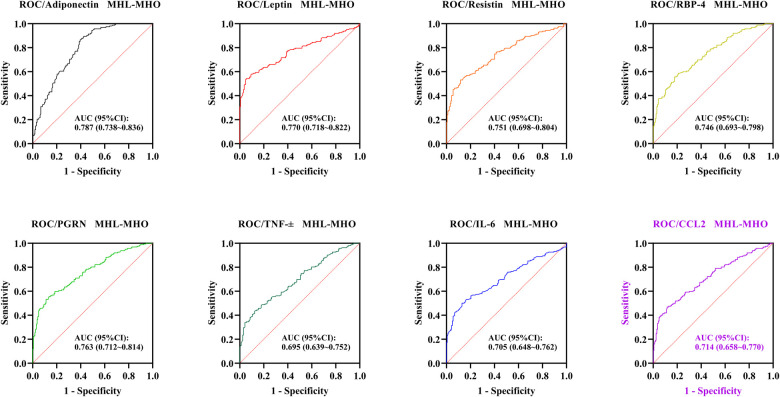
ROC curves of adipose-inflammatory factors for distinguishing MHL from MHO. ROC, Receiver operating characteristic; MHO, Metabolically healthy obesity; MUO, Metabolically unhealthy obesity.

When differentiating MHL from MHO, all adipose-inflammatory factors showed significant discriminatory ability. The AUC (95% CI) values were 0.787 (0.738–0.836) for adiponectin, 0.770 (0.718–0.822) for leptin, 0.751 (0.698–0.804) for resistin, 0.746 (0.693–0.798) for RBP-4, 0.763 (0.712–0.814) for PGRN, 0.695 (0.639–0.752) for TNF-α, 0.705 (0.648–0.762) for IL-6, and 0.714 (0.658–0.770) for CCL2 (all *P* < 0.001) (Table 3, Figure 1).

**Table 3:** Diagnostic efficacy of adipose-inflammatory factors in distinguishing MHL from MHO.

Factor	AUC	95% CI	Youden's index	Sensitivity	Specificity	*P*
Adiponectin	0.787	0.738–0.836	0.470	87.70%	59.30%	<0.001
Leptin	0.770	0.718–0.822	0.488	53.70%	95.10%	<0.001
Resistin	0.751	0.698–0.804	0.414	53.10%	88.30%	<0.001
RBP-4	0.746	0.693–0.798	0.377	55.60%	82.10%	<0.001
PGRN	0.763	0.712–0.814	0.420	55.60%	86.40%	<0.001
TNF-α	0.695	0.639–0.752	0.309	43.20%	87.70%	<0.001
IL-6	0.705	0.648–0.762	0.370	50.00%	87.00%	<0.001
CCL2	0.714	0.658–0.770	0.352	46.90%	88.30%	<0.001

### 3.4 Diagnostic efficacy of adipose-inflammatory factors in distinguishing MHL from MUO

**Figure 2:**
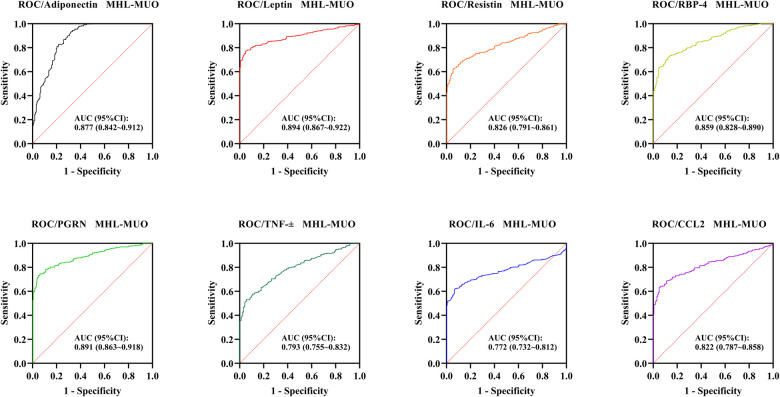
ROC curves of adipose-inflammatory factors for distinguishing MHL from MUO.

Similarly, these adipose-inflammatory factors also demonstrated significant diagnostic performance in distinguishing MHL from MUO. The AUC (95% CI) values were 0.877 (0.842–0.912) for adiponectin, 0.894 (0.867–0.922) for leptin, 0.826 (0.791–0.861) for resistin, 0.859 (0.828–0.890) for RBP-4, 0.891 (0.863–0.918) for PGRN, 0.793 (0.755–0.832) for TNF-α, 0.772 (0.732–0.812) for IL-6, and 0.822 (0.787–0.858) for CCL2 (all *P* < 0.001) (Table 4, Figure 2).

**Table 4:** Diagnostic efficacy of adipose-inflammatory factors in distinguishing MHL from MUO.

Factor	AUC	95% CI	Youden's index	Sensitivity	Specificity	*P*
Adiponectin	0.877	0.842–0.912	0.611	93.20%	67.90%	<0.001
Leptin	0.894	0.867–0.922	0.723	77.20%	95.10%	<0.001
Resistin	0.826	0.791–0.861	0.565	62.70%	93.80%	<0.001
RBP-4	0.859	0.828–0.890	0.599	72.20%	87.70%	<0.001
PGRN	0.891	0.863–0.918	0.684	74.60%	93.80%	<0.001
TNF-α	0.793	0.755–0.832	0.474	53.00%	94.40%	<0.001
IL-6	0.772	0.732–0.812	0.550	61.80%	93.20%	<0.001
CCL2	0.822	0.787–0.858	0.581	63.00%	95.10%	<0.001

### 3.5 Diagnostic efficacy of adipose-inflammatory factors in distinguishing MHO from MUO

**Figure 3:**
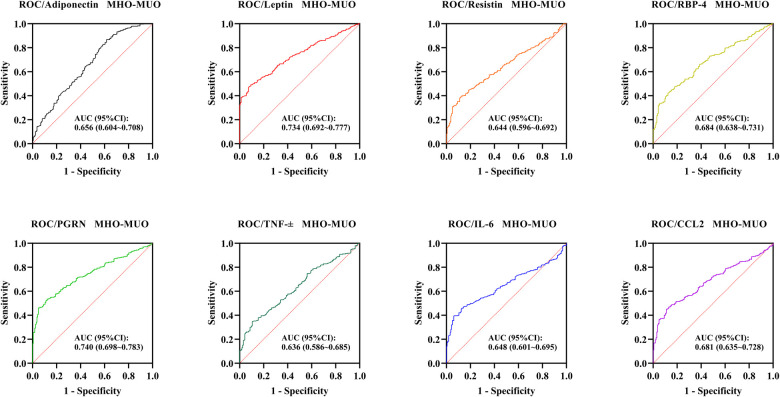
ROC curves of adipose-inflammatory factors for distinguishing MHO from MUO.

For the discrimination between MHO and MUO, all adipose-inflammatory factors remained statistically significant, although their overall diagnostic performance was relatively modest. The AUC (95% CI) values for distinguishing MHO from MUO were 0.656 (0.604–0.708) for adiponectin, 0.734 (0.692–0.777) for leptin, 0.644 (0.596–0.692) for resistin, 0.684 (0.638–0.731) for RBP-4, 0.740 (0.698–0.783) for PGRN, 0.636 (0.586–0.685) for TNF-α, 0.648 (0.601–0.695) for IL-6, and 0.681 (0.635–0.728) for CCL2 (all *P* < 0.001) (Table 5, Figure 3).

**Table 5:** Diagnostic efficacy of adipose-inflammatory factors in distinguishing MHO from MUO.

Factor	AUC	95% CI	Youden's index	Sensitivity	Specificity	*P*
Adiponectin	0.656	0.604–0.708	0.250	86.70%	38.30%	<0.001
Leptin	0.734	0.692–0.777	0.398	49.10%	90.70%	<0.001
Resistin	0.644	0.596–0.692	0.271	38.80%	88.30%	<0.001
RBP-4	0.684	0.638–0.731	0.300	42.30%	87.70%	<0.001
PGRN	0.740	0.698–0.783	0.413	53.60%	87.70%	<0.001
TNF-α	0.636	0.586–0.685	0.241	34.60%	89.50%	<0.001
IL-6	0.648	0.601–0.695	0.331	39.30%	93.80%	<0.001
CCL2	0.681	0.635–0.728	0.339	45.00%	88.90%	<0.001

### 3.6 Prevalence of NAFLD among children with different obesity phenotypes

Among the 162 MHO children, 47 (29.01%) had NAFLD, while 115 (70.99%) did not. Among the 338 MUO children, 156 (46.15%) had NAFLD, while 182 (53.85%) did not. The difference between the two groups was statistically significant (*χ²* = 13.343, *P* < 0.001).

### 3.7 Correlation between adipose-inflammatory factors and NAS in MHO children with NAFLD

**Figure 4:**
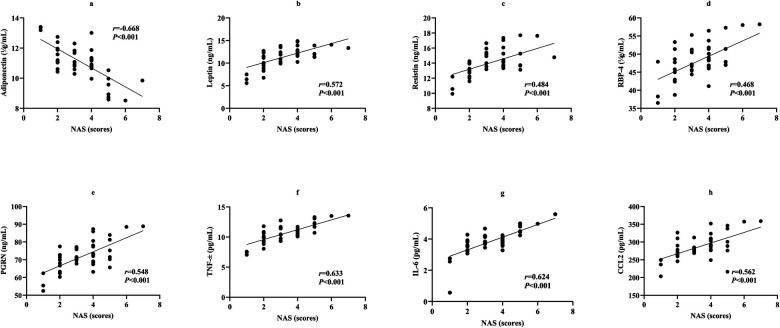
Scatter plots showing correlations between adipose-inflammatory factors and NAS. **(a)**, Adiponectin and NAS; **(b)**, Leptin and NAS; **(c)**, Resistin and NAS; **(d)**, RBP-4 and NAS; **(e)**, PGRN and NAS; **(f)**, TNF-α and NAS; **(g)**, IL-6 and NAS; **h**, CCL2 and NAS. NAS, NAFLD activity score; RBP-4, Retinol-binding protein 4; PGRN, Progranulin; TNF-α, Tumor necrosis factor-alpha; IL-6, Interleukin-6; CCL2, Monocyte chemoattractant protein-1.

Spearman's correlation analysis in the 47 MHO children with NAFLD showed a negative correlation between adiponectin and NAS (*r* = −0.668, *P* < 0.001). Leptin, resistin, RBP-4, PGRN, TNF-α, IL-6, and CCL2 all showed positive correlations with NAS (*r* = 0.572, 0.484, 0.468, 0.548, 0.633, 0.624, 0.562, all *P* < 0.001) (Figure 4).

### 3.8 Correlation between adipose-inflammatory factors and SAF score in MHO children with NAFLD

**Figure 5:**
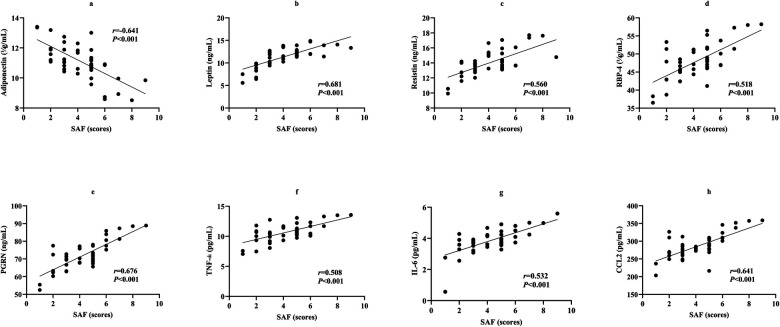
Scatter plots showing correlations between adipose-inflammatory factors and SAF score.

Spearman's correlation analysis in the 47 MHO children with NAFLD showed a negative correlation between adiponectin and SAF score (*r* = −0.641, *P* < 0.001). Leptin, resistin, RBP-4, PGRN, TNF-α, IL-6, and CCL2 all showed positive correlations with SAF score (*r* = 0.681, 0.560, 0.518, 0.676, 0.508, 0.532, 0.641, all *P* < 0.001) (Figure 5).

## 4 Discussion

Research on the metabolic heterogeneity of childhood obesity has progressed from phenotypic description to molecular mechanisms. Adipose-inflammatory factors, serving as a critical bridge connecting energy metabolism and immune regulation, have become a recent focus due to their differential expression patterns across obesity subtypes. With advances in clinical diagnostics, the detection rate of NAFLD in the pediatric population has been increasing annually (up to 36.1%), but consensus on its pathogenesis in MHO children remains elusive (22). Existing evidence suggests that individuals with MHO, despite normal traditional metabolic parameters, may experience covert remodeling of the adipose tissue microenvironment, manifesting as an imbalance in adipokine secretion and early activation of a low-grade inflammatory state. This subclinical alteration may be a potential driver of hepatic steatosis (23). Furthermore, compared to adults, dynamic changes in adipose-inflammatory factors during childhood are more susceptible to influences from developmental stage, dietary patterns, and genetic background, possibly resulting in unique patterns of association with NAFLD progression. Therefore, elucidating the aberrant profile of adipose-inflammatory factors in MHO children and their intrinsic link to liver injury holds significant theoretical value for redefining the concept of metabolic health in childhood obesity and refining the NAFLD risk stratification system.

The results of this study indicated that although MHO children did not exhibit significant glucose or lipid metabolic abnormalities (blood pressure, blood glucose, lipids, etc., although slightly higher than the MHL group, remain within normal ranges), their circulating levels of various adipose-inflammatory factors were already markedly altered. This was characterized by significantly reduced adiponectin levels, alongside elevated levels of leptin, resistin, RBP-4, PGRN, and the inflammatory cytokines TNF-α, IL-6, and CCL2. Furthermore, these levels lay intermediate between those of the MHL and MUO groups. This finding partially aligns with studies in adult MHO populations (24), suggesting that adipose tissue dysfunction may precede abnormalities in traditional metabolic parameters in MHO children. This may be because adipocytes in obese children have relatively strong proliferative and differentiation capacities. Adipose tissue might temporarily maintain metabolic stability through compensatory expansion. However, when long-term energy intake exceeds expenditure, excessive adipocyte recruitment during this compensatory process triggers a series of subclinical changes: First, relative hypoxia in adipose tissue due to inadequate oxygen supply activates hypoxia-inducible factor-1*α*, promoting the expression and release of pro-inflammatory cytokines such as TNF-α and IL-6 (25). Second, enhanced endoplasmic reticulum stress in adipocytes, via the PERK/eIF2*α* pathway, inhibits the synthesis and secretion of adiponectin, a key factor with insulin-sensitizing and anti-inflammatory effects, while upregulating the transcription of the pro-inflammatory factor leptin (26). Third, increased macrophage infiltration (particularly M1 pro-inflammatory macrophages) in adipose tissue, secreting chemokines like CCL2, forms an inflammatory amplification loop, further exacerbating the imbalance of adipose-inflammatory factors (27).

ROC curve analysis in this study further confirmed the core value of adipose-inflammatory factors in distinguishing different metabolic phenotypes. In distinguishing MHL from MHO, adiponectin (AUC = 0.787) and leptin (AUC = 0.770) demonstrated the highest diagnostic efficacy, with Youden's indices of 0.470 and 0.488, respectively, suggesting their potential as sensitive indicators for identifying early metabolic abnormality. Leptin, in particular, emerged as the best single factor for distinguishing MHL from MHO with a specificity of 95.10%. This may be closely related to its central role in energy metabolism regulation. Elevated leptin levels not only reflect increased fat mass but also indicate the early onset of central leptin resistance, a key transition point from metabolic health to metabolic abnormality (28). In distinguishing MHL from MUO, leptin (AUC = 0.894) and PGRN (AUC = 0.891) showed the most prominent diagnostic efficacy, with AUC values approaching 0.90, indicating that when obesity progresses to the metabolically unhealthy stage, changes in these two factors are highly specific. In contrast, traditional inflammatory cytokines like TNF-α (AUC = 0.695) showed lower efficacy in distinguishing MHO from MHL, possibly due to their smaller magnitude of change in early obesity, not yet reaching the threshold sufficient for phenotype discrimination. Notably, even in distinguishing the metabolically more similar MHO from MUO, leptin (AUC = 0.734) and PGRN (AUC = 0.740) maintained moderate-to-good diagnostic value, outperforming other factors. This result suggests that leptin and PGRN may be involved in key regulatory processes underlying the differentiation of metabolic phenotypes in obesity, and their level changes could serve as potential markers for predicting the transition from MHO to MUO.

Regarding NAFLD risk, this study found a prevalence of 29.01% in MHO children. Although lower than the 46.15% in the MUO group, this rate is significantly higher than previously reported rates in normal-weight children (approximately 7.6%) (29). These data suggest that even with normal metabolic parameters, obesity itself places children at a significant risk of liver injury. More importantly, in MHO children with NAFLD, levels of adipose-inflammatory factors showed moderate-to-strong correlations (|*r*| = 0.468–0.681) with the severity of histological liver damage (NAS, SAF score), indicating that these factors are not only involved in the onset of NAFLD but are also significantly associated with disease progression. This aligns with the adipose-liver axis theory in adult studies, where adipose tissue-derived factors directly affect the liver via the portal circulation, regulating lipid deposition, insulin signaling, and inflammation (30, 31). For example, adiponectin can inhibit hepatic lipogenesis and enhance fatty acid oxidation by activating the AMPK pathway; its decline weakens hepatic protection. Leptin can promote liver inflammation and fibrosis via the JAK/STAT pathway. Inflammatory factors such as resistin and TNF-α exacerbate IR and hepatocyte damage through the NF-*κ*B pathway (32–34). This study validates the significant correlations of these factors with NAS and SAF score in MHO children, suggesting that even in the absence of overt metabolic abnormalities, dysregulation of adipose-inflammatory factors may independently drive the onset and progression of NAFLD. This finding deepens the re-evaluation of the benign MHO phenotype: its metabolic health may only represent a state of systemic metabolic compensation, while target organs (like the liver) may already be subject to potential damage.

In summary, children with MHO exhibit dysregulation of adipose-inflammatory factors and an increased risk of NAFLD. Adipose-inflammatory factors, particularly adiponectin and leptin, effectively distinguish metabolic phenotypes of obesity and demonstrate moderate-to-strong correlations with the severity of liver injury in MHO children, suggesting their potential value as targets for early intervention. Compared to previous studies focusing on MUO populations, this study emphasizes that MHO children also warrant attention regarding liver health risks, and adipose-inflammatory factors could serve as early warning indicators and intervention targets. Notably, the histological findings based on liver biopsy not only reveal the molecular characteristics of liver injury in MHO children but also provide crucial biological evidence for advancing the transition of pediatric clinical practice toward non-invasive diagnostic strategies. Currently, imaging techniques represented by shear wave elastography (SWE) and ultrasound-based fat quantification have demonstrated potential for dynamic monitoring as alternatives to biopsy (35, 36). Integrating the key factors identified in this study with these non-invasive imaging parameters holds promise for constructing a multidimensional comprehensive assessment model. This approach could not only provide a non-invasive and precise tool for stratifying liver injury risk in MHO children but also open new avenues for dynamically monitoring disease trajectories and evaluating intervention efficacy. Based on this rationale, future research could conduct longitudinal cohort studies to dynamically track the evolution of adipokine profiles in MHO children and, in conjunction with non-invasive imaging techniques, investigate the critical turning points in progression to MUO and NAFLD. This would provide evidence-based foundations for developing early intervention strategies targeting adipose-inflammatory factors.

## Data Availability

The original contributions presented in the study are included in the article/Supplementary Material, further inquiries can be directed to the corresponding author/s.
